# Clinical Audit cycle of Mental Health Act (MHA) documentation for patients on section 3 staying 90 days and over in adult wards at Roseberry park hospital

**DOI:** 10.1192/bjo.2021.904

**Published:** 2021-06-18

**Authors:** Rohini Ravishankar, Raj Kumar, Ramanand Badanapuram

**Affiliations:** Tees, Esk and Wear Valleys NHS Foundation Trust

## Abstract

**Aims:**

To complete the audit cycle on compliance of MHA documentation (including MCA1 form at admission and 3 months, T2 form, SOAD request and T3 form authorization) on patients on section 3 staying 90 days and over in adult wards at Roseberry park hospital

**Method:**

In the initial audit, we collected data from all inpatients on section 3 staying 90 days and over, in Adult acute and rehab wards on Roseberry park hospital between the time period 28/10/19–04/11/19. Using a designated audit data collection tool, information was gathered from each patient's electronic record pertaining to the standards. The same method was used in re-audit where data were collected from all inpatients on section 3 staying 90 days and over in Adult acute wards on Roseberry park hospital between the time period 04/11/20–11/11/20. To note, the rehab ward at Roseberry park hospital was closed in Feb 2020. The data were analysed by the project lead.

**Result:**

In the initial audit, 16 patients records were identified as meeting criteria,out of these 7 (44%) patients were on acute wards and 9 (56%) at rehab ward. Where as in re-audit 5 patients records were identified as meeting criteria and all were on acute wards. Days in Hospital - Ranged from 120 days to 664 days, average being 295 days and median of 186 days in the initial audit compared to121 days to 290 days, average being 170 days and median of 150 days in the reaudit. Percentage of patients records with documented capacity assessment at admission and 3 months were same at 80% and 60% respectively in both audits.T2 form was completed in all consenting patients in both audits. SOAD request sent was recorded in only 1 (25%) patient in the reaudit, which was lower than the initial audit, where in SOAD request was sent in 7 (78%) patients but recorded in 5 (56%) of them. For patients lacking capacity, T3 form was documented only in 4 (45%) patients but T3 form authorisation was discussed with patient and evidenced in case notes in only 1(11%) case in the initial audit, where as in reaudit T3 form was not documented or discussed for any patient.

**Conclusion:**

There needs to be improvement in MHA documentation for detained patients.

